# New Perspectives in the Antimicrobial Activity of the Amphibian Temporin B: Peptide Analogs Are Effective Inhibitors of *Candida albicans* Growth

**DOI:** 10.3390/jof7060457

**Published:** 2021-06-07

**Authors:** Anant Kakar, Jeanett Holzknecht, Sandrine Dubrac, Maria Luisa Gelmi, Alessandra Romanelli, Florentine Marx

**Affiliations:** 1 Biocenter, Institute of Molecular Biology, Medical University of Innsbruck, A-6020 Innsbruck, Austria; anant.kakar@student.i-med.ac.at (A.K.); jeanett.holzknecht@i-med.ac.at (J.H.); 2 Department of Dermatology, Venerology and Allergy, Medical University of Innsbruck, A-6020 Innsbruck, Austria; sandrine.dubrac@i-med.ac.at; 3 Department of Pharmaceutical Sciences, University of Milan, I-20133 Milano, Italy; marialuisa.gelmi@unimi.it

**Keywords:** Temporin B peptide analogs, antifungal, fungicidal activity, *Candida albicans*, keratinocytes, reconstructed epidermis model, skin irritation, IL-1α

## Abstract

Temporin B (TB) is a short, positively charged peptide secreted by the granular glands of the European frog *Rana temporaria*. While the antibacterial and antiviral efficacy of TB and some of its improved analogs are well documented, nothing is known about their antifungal potency so far. We dedicated this study to characterize the antifungal potential of the TB analog TB_KKG6K and the newly designed D-Lys_TB_KKG6K, the latter having the L-lysines replaced by the chiral counterpart D-lysines to improve its proteolytic stability. Both peptides inhibited the growth of opportunistic human pathogenic yeasts and killed planktonic and sessile cells of the most prevalent human pathogen, *Candida albicans.* The anti-yeast efficacy of the peptides coincided with the induction of intracellular reactive oxygen species. Their thermal, cation, pH and serum tolerance were similar, while the proteolytic stability of D-Lys_TB_KKG6K was superior to that of its template peptide. Importantly, both peptides lacked hemolytic activity and showed minimal in vitro cytotoxicity in primary human keratinocytes. The tolerance of both peptides in a reconstructed human epidermis model further supports their potential for topical application. Our results open up an exciting field of research for new anti-*Candida* therapeutic options based on amphibian TB analogs.

## 1. Introduction

Temporins belong to one of the largest families of antimicrobial peptides (AMPs) and were originally isolated from the mucus secreted from granular skin glands of the European frog *Rana temporaria.* They represent the first line of defense of the amphibian immune system to fight microbial infections [1]. These biomolecules are mildly cationic (0 to +3 at pH 7) and short (8–14 amino acids (aa) in length), which render them highly attractive candidates for the development of novel antimicrobials, as their activity can be optimized by minor alterations of their primary structure [2,3].

The membrane-active peptide Temporin B (TB; aa sequence: LLPIVGNLLKSLL; molecular weight (MW) 1392.8 Dalton (Da) was studied for its bactericidal activity against Gram-positive bacteria with effective concentrations in the micromolar (μM) range [4]. Efforts were made to improve the activity of this AMP against Gram-negative bacteria using an alanine scan in combination with the addition of two extra lysine residues at the N-terminus. This resulted in the peptide analog TB_KKG6A (aa sequence: **KK**LLPIV**A**NLLKSLL; MW 1663.2 Da) with an elevated positive net charge (from +0.9 to +4, pH 7), and a changed amphipathic profile and helical content, all of which significantly improved the antimicrobial activity against Gram-negative and Gram-positive bacteria and decreased the hemolytic activity [5,6]. Further exchanges of aa that were assumed to be involved in hydrophobic interactions with bacterial lipopolysaccharides resulted in the new analog TB_KKG6K (aa sequence: **KK**LLPIV**K**NLLKSLL; MW 1718.2 Da) that exhibited an even higher antibacterial potency than the template peptide TB_KKG6A [7]. 

Previous studies on the activity of TB analogs have focused primarily on their antibacterial and antiviral efficacy [6,7,8,9,10,11], while the potential of TB and its analogs against fungi was less intensively investigated. Opportunistic fungal pathogens are gaining major attention in medical, pharmaceutical and basic science, as the incidence of fungal infections is rapidly increasing. Currently, over a billion people are affected by fungal infections and more than 1.5 million deaths are caused by fungal diseases annually [12,13]. Fungi are eukaryotes and only a limited number of licensed antifungals are available that target cellular structures that are unique in fungi and are therefore tolerated by the host. The intensive use combined with a fungistatic mode of action provoke a highly selective pressure against some of these drugs, and the development of resistance mechanisms in fungi pose a severe impact on global health [14]. 

The number of infections by *Candida* spp. is raising dramatically, whereby *Candida albicans* is the most prevalent opportunistic human pathogenic yeast [15,16]. Residing in most individuals as a harmless commensal, *C. albicans* overgrowth can be triggered by certain conditions that compromise the health balance, e.g., alteration of the host microbiota due to medication, microbial infections or diseases and therapies that impair the immune system [17]. *C. albicans* can cause recurring superficial mucosal and dermal infections or invade deeper tissues and evoke systemic mycoses associated with high mortality [18,19]. Thus, the search for new antifungal drugs and the development of new treatment strategies with improved host tolerance has become a priority objective in the last decades.

In this study, we report for the first time on the antifungal potency of the TB peptide analog TB_KKG6K and a variant thereof, in which the L-lysines were replaced by the chiral counterpart D-lysines (D-Lys_TB_KKG6K) to improve the peptide’s proteolytic tolerance. For the characterization of their antifungal potency, we applied a combination of physicochemical, microbiological and cell biological analyses, and provided a proof-of-principle for their tolerance on human skin cells. Our results promise a high potential for the topical application of these amphibian biomolecules in a clinical setting.

## 2. Materials and Methods

### 2.1. Microorganisms, Media and Growth Conditions

The microorganisms and culture media used in this study are listed in Tables S1 and S2, respectively. Single colonies of *Candida* spp. grown on potato dextrose agar (PDA) were used to inoculate 10 mL of 5% potato dextrose broth (0.05 × PDB). After overnight cultivation at 30 °C and shaking at 200 rpm, the cells were counted and diluted in 0.05 × PDB to the cell number applied in the respective experiments. For the cultivation of *Staphylococcus aureus*, a single colony grown on tryptic soy agar (TSA) was used to inoculate 3 mL of tryptic soy broth (TSB) and incubated overnight at 37 °C with shaking at 200 rpm. A 1:100 dilution in TSB was grown until the culture reached an optical density (OD) of 0.5 at 620 nm (OD_620_), assuming that an OD_620_ of 1 correlates with 8 × 10^8^ bacterial cells mL^−1^. The cell number was adjusted to 1 × 10^4^ mL^−1^ in TSB for use in broth microdilution assays.

### 2.2. Peptide Synthesis

The TB peptides were synthesized on solid phase, using standard protocols for the Fmoc chemistry and were then purified by reversed phase–high performance liquid chromatography (RP–HPLC) and analyzed by electrospray ionization–mass spectrometry (ESI–MS; Supplementary Materials, Methods).

### 2.3. Circular Dichroism Spectroscopy

Circular dichroism (CD) spectroscopy analysis was performed on a Jasco J-810 instrument (Hachioji, Tokyo, Japan). The peptides’ concentration was measured at 205 nm using a molar extinction coefficient of 39,320 M^−1^ cm^−1^. CD spectra were recorded in the 260–203 nm range, at 25 °C and 50 nm min^−1^ scan speed; band width: 1 nm; response: 2 s; and were reported in molar ellipticity. Peptides were dissolved in 10 mM phosphate-buffered saline (PBS, pH 7.4) or 10 mM PBS (pH 7.4)/10 mM sodium dodecyl sulfate (SDS).

### 2.4. Broth Microdilution Assays 

The inhibitory concentration of the TB peptide analogs, defined as the concentration that reduces microbial growth ≥ 90% (IC_90_), was determined for microorganisms using broth microdilution assays carried out in 96-well microtiter plates (Nunclon Delta, Thermo Fisher Scientific, Waltham, MA, USA), as described by Holzknecht et al. [20]. One hundred μL of *Candida* spp. (1 × 10^4^ mL^−1^ in 0.05 × PDB) were mixed with 100 μL of two-fold peptide dilutions in 0.05 × PDB and incubated at 30 °C for 24 h under static conditions. Serial dilutions of the standard antifungal drug Amphotericin B (4.0–0.015 µg mL^−1^) were tested with *C. albicans*. The same assay conditions were applied with *S. aureus*, except for the test medium, where TSB was used, and the incubation temperature, which was 37 °C. To investigate the tolerance to high temperature (95 °C), pH 1.5 and pH 11, proteolysis by trypsin, chymotrypsin and proteinase K, 1.25–5% (*v*/*v*) fetal calf serum (FCS) and the salts CaCl_2_ (0.75–3 mM), MgCl_2_ (1.5–6 mM), NaCl (50–200 mM) and KCl (3–12 mM), the TB peptide analogs were exposed to respective experimental conditions and the IC_90_ was determined against *C. albicans* (Supplementary Materials, Methods). For the evaluation of microbial growth in the broth microdilution assays, the microorganisms were resuspended by vigorous pipetting and the OD_620_ was measured with a multimode microplate reader (FLUOstar Omega, BMG Labtech, Ortenberg, Germany). The OD_620_ of the untreated control was assigned 100% growth. All samples were prepared in technical triplicates and the assays were repeated at least twice. 

### 2.5. Determination of the Fungicidal Activity

*C. albicans* (1 × 10^4^ cells mL^−1^) was incubated with the TB peptide analogs at concentrations corresponding to 0.25 × and 1 × IC_90_, respectively, in 0.05 × PDB at 30 °C under continuous shaking (800 rpm) in a Thermomixer Comfort (Eppendorf, Hamburg, Germany). At the time points 5 min, 6 h and 24 h, aliquots were taken and plated in appropriate dilutions onto PDA plates in duplicates to stimulate the growth of viable cells. The colony-forming units (CFU) were counted after incubation at 30 °C for 24 h. The survival rate was calculated by comparing the number of CFU at the different IC_90_ concentrations with that of the untreated control (representing 100% growth) at each of the defined time points. To determine the efficacy of the TB peptide analogs on sessile *C. albicans* cells, biofilm formation was induced by adding 100 μL of *C. albicans* (1 × 10^6^ cells mL^−1^) in 0.05 × PDB to each well of a 96-well, flat-bottom microtiter plate (Nunclon Delta, Thermo Fisher Scientific, Waltham, MA, USA) and incubating for 24 h at 30 °C. The formed biofilm was then evaluated microscopically for cell attachment and pseudo-hyphae formation before proceeding with peptide application. The serially diluted TB peptide analogs (in 0.05 × PDB) were added to the biofilm in 100 μL aliquots to reach a final volume of 200 μL per well. For the positive control, 10 µg mL^−1^ Amphotericin B (Sigma-Aldrich, St. Louis, MO, USA) was used, while 0.05 × PDB was added to the growth control. After further incubation for 24 h at 30 °C, the biofilm was disrupted by vigorous pipetting, and 20 µL of the detached cells were mixed with 80 µL of 0.05 × PDB. Appropriate dilutions were then plated in duplicates onto PDA to stimulate the growth of survivor cells. The effectiveness of the TB peptide analogs to reduce biofilm formation was calculated by comparing the number of CFU at different peptide concentrations with that of the untreated growth control. All samples were plated in duplicates and the assays were repeated at least twice.

### 2.6. Analysis of the Induction of Intracellular Reactive Oxygen Species and Cell Death 

The induction of intracellular reactive oxygen species (iROS) in *C. albicans* in response to the TB peptide analog exposure was detected with a fluorometric assay using the fluorogenic dye 2’,7’-dichlorofluorescin diacetate (DCFH-DA; Sigma-Aldrich, St. Louis, MO, USA), as described by Sonderegger et al. [21]. The DCFH-DA stock solution (100 mM in DMSO) was added to the *C. albicans* cells (1 × 10^7^ mL^−1^) at a final concentration of 100 μM. The samples were pre-incubated for 30 min at 30 °C in the dark. Serial dilutions of the TB peptide analogs were prepared in 0.05 × PDB in 96-well microtiter plates (Nunclon Delta, Thermo Fisher Scientific, Waltham, MA, USA) in a final volume of 100 μL per well. H_2_O_2_ (Sigma-Aldrich, St. Louis, MO, USA) was used as a positive control for iROS induction. The DCFH-DA-loaded cells (100 μL per well) were added to the test compounds to give a final volume of 200 μL per well. To prove the peptide specific iROS induction, 50 µL of ascorbic acid (1 mg mL^−1^ in H_2_O; Sigma-Aldrich, St. Louis, MO, USA) were added to the DCFH-DA-loaded cells, resulting in an antioxidant concentration of 200 µg mL^−1^ in a total volume of 250 µL per well. The 96-well plate was immediately placed in a multimode microplate reader (FLUOstar Omega, BMG Labtech, Ortenberg, Germany) with a pre-set incubation temperature of 30 °C. The dichlorodihydrofluorescein (DCF) fluorescence signal was recorded every 5 min over a period of 4 h at excitation and emission wavelengths of 485 and 520 nm, respectively. All samples were prepared in triplicates and the experiments were conducted at least twice. The cell death induction by TB peptide analogs was assessed with propidium iodide (PI; Sigma-Aldrich, St. Louis, MO, USA) staining, as described in the Supplementary Materials, Methods.

### 2.7. Hemolytic, Cytotoxic and Skin Irritation Potential of the TB Peptide Analogs

Assays to evaluate the induction of hemolysis and the metabolic inactivation of primary human keratinocytes by peptide exposure are described in the Supplementary Materials, Methods. The TB peptide analogs were tested for their skin irritation potential in vitro using the three-dimensional (3D) Phenion^®^ open-source reconstructed epidermis model (Henkel, Düsseldorf, Germany) with slight adaptation from Groeber et al. and Mewes et al. [22,23] and the test guidelines 439 of the Organization for Economic Cooperation and Development (OECD) [24]. In brief, the epidermal tissue models were equilibrated in 6-well plates (STARLAB, Hamburg, Germany) with 1.5 mL of Phenion^®^ OS_REp air-liquid interface (ALI) medium (Henkel, Düsseldorf, Germany) for 24 h at 37 °C, 5% (*v*/*v*) CO_2_. Then, the medium was replaced by fresh medium and 25 µL Dulbecco’s phosphate-buffered saline (D-PBS) containing 50 μg of the peptide, respectively, was dispensed directly on top of the models and spread gently over the surface with sterile glass applicators. The same volume of D-PBS and of 5% (*w*/*v*) aqueous SDS were applied on the negative and positive control models, respectively. All samples and controls were prepared in triplicates. After incubation at 25 °C for 35 min, each model was thoroughly dipped six times in 1 L of D-PBS. The models were transferred into 6-well plates with 1.5 mL of Phenion^®^ OS_REp ALI medium (Henkel, Düsseldorf, Germany) and incubated for another 24 h (37 °C, 5% (*v*/*v*) CO_2_). The culture medium was collected and stored at −80 °C until required for cytokine quantification, which was performed according to the manufacturer’s instructions (Supplementary Materials, Methods). For metabolic activity testing, the models were transferred to 24-well plates (STARLAB, Hamburg, Germany) containing 0.2 mL of 3-[4,5-dimethylthiazol-2-yl]-2,5-dipheyltetrazolium bromide (MTT; Sigma-Aldrich, St. Louis, MO, USA) solution (1 mg mL^−1^ in D-PBS) and were further incubated for 3 h (37 °C, 5% (*v*/*v*) CO_2_). Then, they were transferred into a new 24-well plate, and the insoluble blue formazan crystals that had formed by intracellular MTT reduction in metabolically active cells were extracted with 2 mL of 2-propanol [25]. After further 2 h of incubation at 25 °C and shaking at 250 rpm, a sharp cannula was used to prick the insert membrane so that the tissue fluid mixed with the fluid in the wells. From each tissue sample, 200 µL of the formazan extraction solution were transferred in duplicates to a 96-well microtiter plate and the optical density was measured in a multimode plate reader (FLUOstar Omega, BMG Labtech, Ortenberg, Germany) at a wavelength of 550 nm. The relative metabolic activity was calculated from two replicates per tissue and three tissues per assay. The values are given as the percentage of the mean OD_550_ (mean ± SD) of the negative control (treated with D-PBS), which was assigned a value of 100%. 

### 2.8. Statistics

Data analysis was conducted with Microsoft Excel software (2016, Version 16.16.16; Microsoft Corp., Albuquerque, NM, USA). Values are given as mean ± SD. For the calculation of significant differences between the data obtained from treated samples vs. untreated controls, a two-tailed, two-sample Student’s *t*-test was applied. *p*-values of ≤0.05 were considered as significant, and *p*-values of ≤0.005 were considered as highly significant. 

### 2.9. Ethics Statement

The study was approved by the Ethics Committee of the Medical University of Innsbruck and conducted in accordance with the Declaration of Helsinki principles. All study subjects gave written informed consent and participated voluntarily. Biopsies were taken from non-UV-exposed trunk skin of adult European healthy control subjects (AN 5073 325/4.2 360/5.8 (3804a): 2013-2017; AN 2016-0260 368/4.22 421/AM1 (4537a): 2016-2025).

## 3. Results

### 3.1. Conformation of TB Peptide Analogs

**Figure 1:**
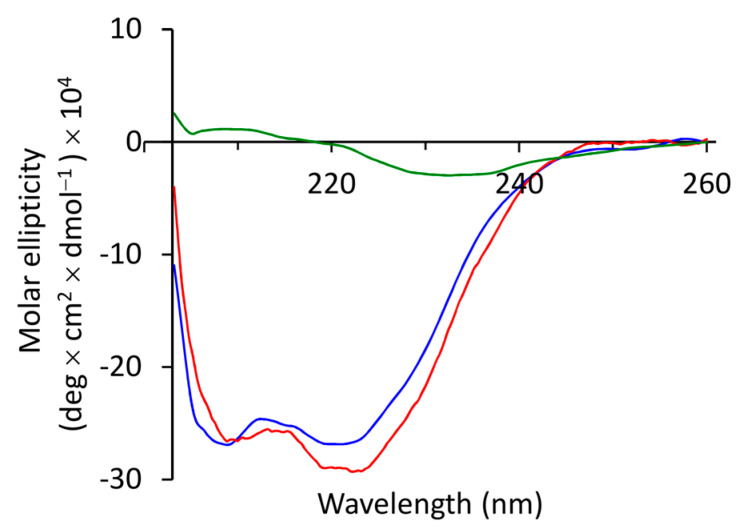
CD spectra of TB peptide analogs. CD spectra of TB_KKG6K (blue), D-Lys_TB_KKG6K (red) and TB_KKG6K^scrambled^ (green), acquired in 10 mM PBS/10 mM SDS (pH 7.4).

After synthesis by Fmoc chemistry and purification by RP-HPLC (Figure S1), the TB peptides were analyzed by ESI–MS, which revealed the following molecular masses in Da: TB_KKG6K 1719.23 [M+H]^+^, 859.92 [M+2H]^2+^, 573.82 [M+3H]^3+^ and D-Lys_TB_KKG6K 1719.84 [M+H]^+^, 860.43 [M+2H]^2+^, 574.04 [M+3H]^3+^. The detected mass of the peptides corresponded to the calculated theoretical mass of 1718.18 Da, respectively. To investigate the role of the primary structure and charge distribution on the peptide’s conformation and antimicrobial activity, we synthesized a peptide with a scrambled aa sequence, TB_KKG6K^scrambled^, in which all L-lysines were positioned at the peptide’s N-terminus (aa sequence: KKKKLLPIVNLLSLL; detected MS: 1719.80 [M+H]^+^, 860.30 [M+2H]^2+^, 574.02 M+3H]^3+^; calculated mass of 1718.18 Da). The secondary structure of the peptides was analyzed with CD spectroscopy either in 10 mM PBS (pH 7.4), or in 10 mM PBS/10 mM SDS (pH 7.4). The latter conditions were chosen to mimic the negatively charged surface of fungal membranes. Neither of the peptides adopted a defined structure in PBS, as expected (Figure S2). In the presence of SDS, however, the CD spectra of TB_KKG6K and D-Lys_TB_KKG6K showed two minima at 208 nm and 222 nm, suggesting that both peptides existed in an α-helical conformation (Figure 1). In contrast, the CD spectrum of TB_KKG6K^scrambled^ exhibited a weak minimum at 230 nm, which is characteristic for peptides that aggregate in β-sheet structures [26].

### 3.2. Antimicrobial Activity of the TB Peptide Analogs

The IC_90_ of the TB peptide analogs was determined against the opportunistic human pathogenic yeasts *C. albicans*, *Candida glabrata* and *Candida parapsilosis* in broth microdilution assays (Table 1). In addition, the Gram-positive bacterium *S. aureus* was included as a TB-sensitive control strain. TB_KKG6K and D-Lys_TB_KKG6K were similarly effective and inhibited the growth of all *Candida* spp., including the fluconazole-resistant clinical isolate 27700 [27] at an IC_90_ of 1.8 µM (Table 1). For comparison, the licensed antifungal drug Amphotericin B inhibited the growth of *C. albicans* at the IC_90_ of 0.06 µM (0.055 µg mL^−1^). Interestingly, the variant TB_KKG6K^scrambled^ showed the same efficacy against *C. albicans* as the other two TB peptide analogs, which suggests that the physicochemical properties determine the antifungal activity of the peptides, while their primary structure and conformation seem to be of less importance (Table 1). All TB analogs inhibited the growth of *S. aureus,* but higher concentrations were necessary to limit bacterial rather than fungal growth, with TB_KKG6K being more effective (IC_90_ 3.6 µM) than TB_KKG6K^scrambled^ (IC_90_ 7.2 µM) and D-Lys_TB_KKG6K (IC_90_ 14.4 µM) (Table 1). In the following study, we concentrated on the characterization of the antifungal activity of TB_KKG6K and D-Lys_TB_KKG6K.

**Figure 2:**
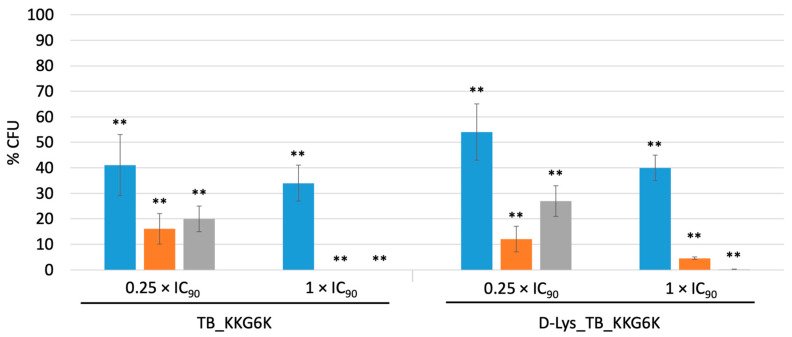
Candidacidal activity of the TB peptide analogs. *C. albicans* cells were exposed to 0.25 × IC_90_ and 1 × IC_90_ of TB_KKG6K and D-Lys TB_KKG6K, respectively, for 5 min (blue), 6 h (orange) or 24 h (grey). Appropriate dilutions of the samples were plated onto PDA plates and the number of CFU were determined after 24 h of incubation at 30 °C. CFU counts without peptide treatment were assigned a value of 100% (untreated control). Values represent the mean ;± SD (n = 3). Significant differences between data of treated samples and the untreated control are indicated; ** *p* ≤ 0.005.

To test the TB peptide analogs for their fungicidal potential, *C. albicans* cells were incubated with the peptides at 0.25× and 1 × IC_90_ for 5 min, 6 h and 24 h, respectively, and appropriate dilutions were plated onto PDA plates to quantify the number of cells that resumed growth after further 24 h of incubation by counting the CFU. The CFU of the untreated control at each time point was assigned 100%. Figure 2 shows that both peptides significantly reduced the CFU counts in a concentration- and time-dependent manner. At 1 × IC_90_, the TB_KKG6K reduced the viability by 66% shortly after peptide addition to the *Candida* cells and reached 100% killing after 6 h of incubation. The same concentration of D-Lys_TB_KK6GK had a similar but delayed killing effect (100% killing after 24 h). Sublethal concentrations of the peptides (0.25 × IC_90_) resulted in a less efficient killing activity, and a slight increase in the CFU number between 6 h and 24 h of peptide exposure pointed at the resumption of fungal growth, indicating a fungistatic mode of action at this low concentration.

Next, we investigated the efficacy of the TB analogs to inhibit the growth of sessile *Candida* cells. To this end, a 24 h old biofilm was exposed to the peptides and the % CFU were evaluated with a plating assay. Both TB analogs significantly reduced the number of surviving cells at a concentration 5 × higher than the IC_90_ (9 µM) for planktonic cells and effectively killed sessile cells at a 10 × higher IC_90_ concentration (18 µM), showing a similar efficacy in biofilm reduction as 10 mg mL^−1^ Amphotericin B, which was included in the test as a positive control (Table 2).

### 3.3. Tolerance of the TB Peptide Analogs to High Temperature, Extreme pH, Proteolytic Degradation, Serum and Cations

The ability of AMPs to withstand harsh environmental conditions such as high temperature, extreme pH, proteolytic degradation, serum compounds, and high cation concentrations are important prerequisites for their commercial feasibility as novel antimicrobial agents. The TB peptide analogs were tested for each condition and their IC_90_ was evaluated by applying them post-exposure in broth microdilution assays with *C. albicans*. As summarized in Table 3, the 1 h treatment at 95 °C increased the IC_90_ of both peptides by only one dilution step, and the exposure to pH 1.5 and pH 11 minimally affected the antifungal activity of D-Lys_TB_KKG6K. Expectedly, the D-lysines rendered this peptide variant more resistant against proteolysis by trypsin, chymotrypsin and proteinase K, all of which readily inactivated TB_KKG6K (Table 3). To avoid interference of the proteases themselves on the growth of *C. albicans*, chymotrypsin and proteinase K were thermally inactivated (95 °C for 15 min) and trypsin was neutralized by the addition of 1.25% (*v*/*v*) FCS before the protease-peptide samples were applied in the tests. The thermal treatment and the presence of FCS had no adverse effect on both peptides’ activity (Table 3 and Table S3).

Furthermore, the activity of the TB peptide analogs against *C. albicans* was evaluated in the presence of 1.25–5% FCS, 50–200 mM NaCl, 3–12 mM KCl, 0.75–3 mM MgCl_2_ and 1.5–6 mM CaCl_2_ (Table 4). Both peptides retained their antifungal potential in the presence of 3–12 mM KCl, 0.75 mM MgCl_2_ and 1.25% FCS. The peptides were most sensitive to NaCl and CaCl_2_, whereby no IC_90_ could be determined when the test medium was supplemented with 6 mM CaCl_2_. D-Lys_TB_KKG6K was more sensitive, showing, in general, IC_90_ values which were twofold higher in the presence of cations than those obtained with TB_KKG6K (Table 4).

### 3.4. The Induction of iROS Species by D-Lys_TB_KKG6K

**Figure 3:**
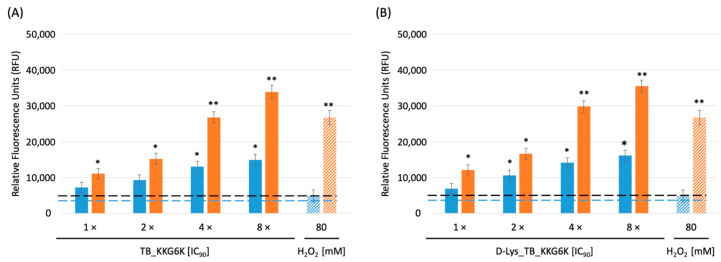
Induction of iROS in *C. albicans* after 1 h (blue bars) and 4 h (orange bars) of treatment with TB-KKG6K and D-Lys_TB_KKG6K. The fluorescent DCF signal is given in RFU. The dashed blue line represents the background RFU (3682 ± 284) at the 1 h time point, the dashed black line the RFU (4798 ± 391) at the 4 h time point of the untreated, negative control, respectively. As a positive control, 80 mM of the iROS inducer H_2_O_2_ (patterned bars, extreme right in (**A**) and (**B**), respectively) was used. Values represent mean ± SD (n = 3). Significant differences between data of the treated samples and the negative control are indicated; * *p* ≤ 0.05 and ** *p* ≤ 0.005.

Many AMPs were reported to kill microorganisms by inducing iROS [21,29,30]. Therefore, we analyzed the iROS inducing potential of the TB peptide analogs by the use of the fluorogenic dye DCFH-DA that is converted intracellularly to the fluorescent DCF when oxidized in the presence of iROS [31]. Both peptides induced iROS at a similar level in a time- and concentration-dependent manner. Figure 3 shows the iROS burden (represented by relative fluorescence units RFU) in *C. albicans* exposed for 1 h and 4 h to 1×–8 × IC_90_ of the TB peptide analogs, respectively. At both time points, peptide concentrations ≥ 4 × IC_90_ induced higher iROS levels than 80 mM H_2_O_2_, which was used as an iROS inducing positive control. The untreated cells (negative control) showed low RFU levels at the respective time points (Figure 3).

The addition of 1 mM ascorbic acid to the test system prevented the induction of iROS in *C. albicans*, as shown for D-Lys_TB_KKG6K (Figure S3A). Concomitantly, the induction of plasma membrane permeabilization and cell death was prevented, as visualized with PI (Figure S3B). This fluorescent dye is membrane impermeable, but is internalized and binds to nucleic acids when the plasma membrane is permeabilized, which is an indicator of cell death. 

### 3.5. Analysis of the Impact of D-Lys_TB_KKG6K on Mammalian Cells In Vitro

The reported hemolytic and cytotoxic activity of many membrane-active, cationic AMPs on mammalian cells hamper their use as antimicrobial drugs [32,33]. For the TB peptide analog TB_KKG6A no hemolytic activity was detected [5]. Here we applied a basic disk diffusion assay to qualitatively assess the peptides’ hemolytic potential. The improved analogs TB_KKG6K and D-Lys_TB_KKG6K did not induce the hemolysis of sheep erythrocytes in Columbia blood agar plates when applied at the very high amount of 50 μg per disk (Figure S4). The sensitivity of the TB peptide analogs towards some cations hampers their applicability for systemic administration, though they could be feasible for the development of drugs for topical application. To explore their suitability, we evaluated the cytotoxic potential of the TB peptide analogs on primary human keratinocytes in vitro using the colorimetric 2,3-bis-(2-methoxy-4-nitro-5-sulfophenyl)-2H-tetrazolium-5-carboxanilide (XTT) test (Supplementary Materials, Methods). Neither of the peptides affected the metabolic activity of the keratinocytes at the concentrations tested (3.6–57.6 µM), though at the highest concentration the D-Lys_TB_KKG6K was better tolerated than its chiral counterpart TB_KKG6K (Figure S5). To approach test conditions that mimic the natural setting, we tested the irritation potential of the peptides on the 3D reconstructed epidermis model Phenion^®^ OS-REp that was cultured in an ALI phase, as this has been reported to be a valuable model to detect skin irritation [22,23]. The cellular metabolic activity in response to a 35 min exposure to 50 µg of peptides was validated by the reduction of MTT. The positive control model was exposed to 5% SDS (*w*/*v*), while the negative control was treated with D-PBS. The negative control was assigned a metabolic activity of 100%. As shown in Table 5, both peptides were very well tolerated by the OS-REp model, being far from the irritant categorization threshold of ≤50% metabolic activity compared to the negative control, while 5% SDS (*w*/*v*) was cytotoxic [22,23,34]. In addition, we assessed the release of the proinflammatory cytokine IL-1α into the culture medium after the peptide exposure as a supplementary endpoint to the metabolic activity results. None of the peptides significantly increased the IL-1α concentration in the culture medium over that of the negative control, while the cytokine release was 10-fold higher in the SDS-treated control (Table 5).

## 4. Discussion

In spite of the well-documented antibacterial and antiviral activity of TB and TB peptide analogs, [4,6,7,8,9,10,11] information on their antifungal potential is rather scarce. In this study, we evaluated in detail the antifungal properties of the TB peptide analog TB_KKG6K and its chiral counterpart D-Lys_TB_KKG6K. This modification was rationalized by a variety of studies, which indicated that D-aa peptide isoforms, apart from conferring an increased resistance to proteolytic degradation, exhibit increased antimicrobial efficacy and lower hemolytic activity than their L-aa peptide isoforms [35,36,37,38,39]. In contrast to these reports, we did not detect any elevated efficacy of D-Lys_TB_KKG6K over that of TB_KKG6K against the microorganisms tested in this study. On the contrary, the D-form TB peptide analog was less active against *S. aureus*, while both peptides inhibited the growth of *Candida* spp. with the same efficacy. D-aa enantiomers are known as helix-breakers. In D-Lys_TB_KKG6K two D-lysines are located at the more flexible N-terminus and at positions 6 and 10, relative to their position in the original TB peptide [4]. Although it is likely that changing the chirality of the central lysines at positions 6 and 10 alters the three-dimensional structure of the peptide, no conformational changes were detected by CD. Similarly, the CD spectra of Temporin L analogs containing a single D-aa in their sequence matched those of the L-form peptides, but the biological activity was strongly influenced by these substitutions depending on their position [37]. Thus, we cannot exclude that a minor structural change in D-Lys_TB_KKG6K—possibly detectable by nuclear magnetic resonance measurements—is responsible for the reduction in antibacterial activity of this peptide analog. The concentration of the positively charged lysines at the N-terminus of TB_KKG6K^scrambled^ strongly influenced the peptide’s structure and reduced the anti-bacterial but not the anti-yeast efficacy. We hypothesize that the strength of the interaction with *C. albicans* is determined by the total charge of the peptides, rather than by the structure and the distribution of the charged residues within the sequence. This is supported by the observation that TB_KKG6K^scrambled^ shows a similar antifungal activity as the two other TB analogs, although it exhibited a different conformation in the presence of SDS micelles. Due to the N-terminal localization of all positively charged lysines, it is reasonable to assume that the hydrophobic residues pack one onto the other in an antiparallel fashion whereas the positively charged N-terminus remains exposed in order to maximize the surface of interaction with the negatively charged micelles [26]. An interaction that requires a defined peptide conformation (e.g., ligand-target binding) seems rather unlikely. Differences in the composition, structure and function of the outer layers of fungi and bacteria (cell wall, plasma membrane) should also be considered for a possible interpretation of these data and might explain as well the lack of any cytotoxic and lytic activity in human cultured cells and sheep erythrocytes, respectively. Notably, sheep erythrocytes were reported to be more robust against lysis by AMPs than human erythrocytes [40]. The species-specific mode of action in relation to the peptide structure awaits further investigations in the future. 

The ability of *C. albicans* to aggregate and proliferate in surface-associated biofilms is considered to be an important virulence factor in candidiasis. Biofilms exhibit properties that impact the host’s response to infection, and dispersed cells from biofilms display enhanced pathogenicity [41]. Moreover, aggregated *Candida* cells are often less susceptible to antifungal agents, which limits effective anti-*Candida* treatment [42,43]. Therefore, the development of novel drugs with candidacidal activity, suppression/inhibition of resistance development and tolerance by the host remains a priority objective.

Both TB peptide analogs exhibited fungicidal activity on planktonic and sessile *C. albicans* cells, and the cell death was linked with membrane permeabilization and iROS induction. At present, we cannot conclude whether these detriments are primary or secondary effects of the peptides’ mode of action, but their fungicidal potential is relevant to prevent or delay the development of resistance [44]. The TB peptide analogs were resistant to extremes of temperature and pH and were reasonably tolerant towards KCl, MgCl_2_ and FCS. Expectedly, the D-Lys_TB_KKG6K exhibited higher proteolytic stability than TB_KKG6K. The high sensitivity towards NaCl and CaCl_2_, however, let us assume that the TB peptide analogs would be more suitable for topical rather than systemic administration, representing a promising alternative for the treatment of recurring dermal and mucosal infections [45]. Indeed, both peptides were very well tolerated even at very high concentrations when tested in vitro on primary human keratinocytes isolated from trunk skin biopsies, the D-Lys_TB_KKG6K being slightly better tolerated at very high concentrations than TB_KKG6K. Based on these promising results, we utilized the commercially available Phenion^®^ OS-REp model that consisted of fully differentiated primary human keratinocytes as an alternative to conventionally used submersed skin cell culture or animal models [22,23]. The keratinocytes in the model were cultured under ALI conditions, which helps to promote the differentiation and stratification of the epidermis and facilitates the topical exposure of chemicals onto the *Stratum corneum*, thereby mimicking the application on human skin. An additional advantage of using 3D human epidermal models over animal models, apart from ethical considerations, is the tendency of the latter to result in false positives or negatives, presumably due to the differences between animal and human skin physiology [22,23,46]. Both peptides were equally well tolerated in the tested amounts. According to the OECD guidelines, a decrease of 50% in the cellular metabolic activity compared to the negative control classifies a compound as “irritant” [24], which occurred only with the 5% SDS used as the test substance for irritation. The assessment of the release of the proinflammatory cytokine IL-1α proved to be a sensitive and reliable indicator of cellular insult to validate the results of MTT assays [34,47,48]. Based on our results, both peptides can be categorized as “non-irritant compounds”. It has to be evaluated in future studies if the peptides hold promise in their efficacy to reduce or eradicate the fungal load in full thickness infection models, which possess also a dermal component and can replicate epithelial-mesenchymal signaling in response to diverse stimuli or in appropriate in vivo models [49].

In summary: our work extended the small number of anti-fungal studies on amphibian Temporins and successfully proved the importance of rational peptide modifications to overcome limitations in the parent peptide, which paves the way for the development of new anti-*Candida* therapies.

## Figures and Tables

**Table 1:** Inhibitory concentrations (IC_90_, in µM) of the TB peptide analogs against microorganisms ^§^.

	IC_90_		
Microorganism	TB_KKG6K	D-Lys_TB_KKG6K	TB_KKG6K^scrambled^
*Candida albicans*	1.8	1.8	1.8
*Candida albicans* 27700	1.8	1.8	n.d.
*Candida glabrata*	1.8	1.8	n.d.
*Candida parapsilosis*	1.8	1.8	n.d.
*Staphylococcus aureus*	3.6	14.4	7.2

^§^ The IC_90_ was defined as the peptide concentration that reduces microbial growth by ≥90% compared to the untreated control, which was defined as 100% growth [28]. The IC_90_ was determined against yeasts and bacteria after 24 h of incubation in broth microdilution assays under standard conditions. n.d., not determined.

**Table 2:** Survival of sessile *C. albicans* cells after exposure to the TB peptide analogs for 24 h ^§^.

Treatment	Concentration ^$^	CFU [%]
TB_KKG6K	1.8 (1×)	144.0 ± 17.3 **
	9.0 (5×)	22.8 ± 14.1 **
	18.0 (10×)	2.8 ± 3.3 **
D-Lys_TB_KKG6K	1.8 (1×)	157.1 ± 25.3 **
	9.0 (5×)	19.2 ± 15.5 **
	18.0 (10×)	0.3 ± 0.5 **
Amphotericin B	10.0	0 ± 0 **

^§^ Values of surviving cells are given in % CFU in comparison to the untreated growth control, which was set to be 100%. Values represent the mean ± SD (n = 4). Significant differences between the treated samples vs. the untreated growth control are indicated; ** *p* ≤ 0.005. ^$^ Peptide concentrations are given in μM, corresponding to the ×-fold IC_90_ value determined for planktonic cells in broth microdilution assays (in brackets). As a control, Amphotericin B was included at a concentration of 10 µg mL^−1^.

**Table 3:** Sensitivity of the TB analogs to high temperature, extreme pH and proteolytic degradation ^§^.

	Fold Change in IC_90_	
Applied Condition	TB_KKG6K	D-Lys_TB_KKG6K
**Standard Condition**	1×	1×
**Temperature**		
95 °C, 1 h	2×	2×
**pH**		
1.5	1×	2×
11	1×	2×
**Proteolytic Degradation**		
Trypsin	>4×	1×
Chymotrypsin	>4×	2×
Proteinase K	>4×	2×

^§^ The activity of the TB peptide analogs was evaluated by determining the fold change in their respective IC_90_ values against *C. albicans* in broth microdilution assays after treatment of the peptides under the indicated conditions.

**Table 4:** Fold-change in IC_90_ of TB peptide analogs in the presence of cations and heat-inactivated FCS ^§^.

	Fold Change in IC_90_	
Supplements	TB_KKG6K	D-Lys_TB_KKG6K
**NaCl [mM]**		
200	8×	16×
100	4×	16×
50	2×	4×
**KCl [mM]**		
12	1×	1×
6	1×	1×
3	1×	1×
**CaCl_2_ [mM]**		
6	>8×	>8×
3	4×	8×
1.5	2×	2×
**MgCl_2_ [mM]**		
3	2×	4×
1.5	1×	2×
0.75	1×	1×
**FCS [%]**		
5	4×	4×
2.5	2×	2×
1.25	1×	1×

^§^ The tolerance to cations and FCS of the TB peptide analogs was evaluated by determining the fold-change in the IC_90_ against *C. albicans* in broth microdilution assays in the presence of the indicated supplements.

**Table 5:** Cellular metabolic activity of the in vitro 3D Phenion^®^ OS-REp model and release of proinflammatory cytokine IL-1α in response to the treatment with the TB peptide analogs.

Compound ^#^	Metabolic Activity [%]	IL-1α [pg mL^−1^]	Categorization ^$^
D-PBS	100 ± 4.6	20.6 ± 8.3	non-irritant
5% SDS	1.0 ± 0.1 **	199.2 ± 47.7 **	irritant
TB_KKG6K	94.2 ± 2.9	35.1 ± 32.9	non-irritant
D-Lys_TB_KKG6K	91.4 ± 2.9	41.4 ± 27.8	non-irritant

^#^ The peptides (50 μg) were applied in 25 μL aliquots per model. Values of metabolic activity after peptide treatment were calculated by comparison with the D-PBS treated model (negative irritation control), which was assigned 100% metabolic activity. SDS (5%, *w*/*v*) was used as a positive irritation control (inhibition of metabolic activity). Metabolic activity and IL-1α values represent the mean ± SD (n = 3). ^$^ Irritant categorization threshold ≤ 50% metabolic activity compared to the negative control [24]. Significant differences in IL-1α release between treated samples and negative control (D-PBS treated) is indicated; ** *p* ≤ 0.005.

## Data Availability

Not applicable.

## Supplementary Materials

The following are available online at https://www.mdpi.com/article/10.3390/jof7060457/s1; Table S1: Microorganisms used in this study, Table S2: Media used in this study, Table S3: The impact of protease inactivation conditions on the activity of the TB peptide analogs against *C. albicans*, Figure S1: RP-HPLC profiles of pure TB peptide analogs, Figure S2: CD spectra of TB peptide analogs, Figure S3: Induction of iROS in *C. albicans* after treatment with D-Lys_TB_KKG6K monitored over a time period of 4 h, Figure S4: Hemolytic activity of the TB peptide analogs, Figure S5: Cellular metabolic activity of keratinocytes in response to increasing concentrations of TB peptide analogs over a time period of 6 h.
