# Supplementary material for: New Perspectives in the Antimicrobial Activity of the Amphibian Temporin B: Peptide Analogs Are Effective Inhibitors of Candida albicans Growth
Source: J Fungi (Basel). 2021 Jun 7;7(6):457. doi: 10.3390/jof7060457 (PMC8226839; doi:10.3390/jof7060457)
Supplement: Supplementary file 1 [file jof-07-00457-s001.zip › jof-1211649-supplementary.pdf]

Supplementary Materials

New Perspectives in the Antimicrobial Activity of the Amphibian Temporin B: Peptide Analogs Are Effective Inhibitors of *Candida albicans* Growth

Anant Kakar[1], Jeanett Holzknecht[1], Sandrine Dubrac[2], Maria Luisa Gelmi[3], Alessandra Romanelli[3,*], Florentine Marx[1,*]

[1] Biocenter, Institute of Molecular Biology, Medical University of Innsbruck, A-6020 Innsbruck, Austria; anant.kakar@student.i-med.ac.at (A.K.), jeanett.holzknecht@i-med.ac.at (J.H.)

[2] Department of Dermatology, Venerology and Allergy, Medical University of Innsbruck, A-6020 Innsbruck, Austria; sandrine.dubrac@i-med.ac.at (S.D.)

[3] Department of Pharmaceutical Sciences, University of Milan, I-20133 Milano, Italy; marialuisa.gelmi@unimi.it (M.L.G).

* Correspondence: alessandra.romanelli@unimi.it; Tel. +39-02-5031-4475 (A.R.); florentine.marx@i-med.ac.at; Tel. +43-512-9003-70207 (F.M.)

Keywords: Temporin B peptide analogs; antifungal; fungicidal activity; *Candida albicans*; keratinocytes; reconstructed epidermis model; skin irritation; IL-1 α.

Supplementary Methods

Synthesis of TB peptide analogs

Peptides were synthesized on an automated peptide synthesizer Liberty Blue CEM by standard protocols for Fmoc/tBu chemistry on a 0.1 mmol scale, using the Rink Amide resin. At the end of the synthesis, the peptides were cleaved off the resin and protecting groups were removed by treatment of the resin with a solution of TFA/TIS/H_2O 95/2.5/2.5 (*v/v/v*) for 2 h. Peptides were precipitated in cold ethyl ether, dissolved in water and then lyophilized. Peptides were purified by RP-HPLC, monitoring the absorbance at 210 nm on a Jupiter 10μ Proteo 90A° (100 × 21, 20 mm) column flow rate 20.0 mL min^{-1}; purification of TB_KKG6K and TB_KKG6K^{scrambled} was performed using a gradient of CH_3CN (0.1% TFA) in H_2O (0.1% TFA) from 30 to 80 % in 20 min, purification of D-Lys_TB_KKG6K was performed with a gradient of CH_3CN (0.1% TFA) in H_2O (0.1% TFA) from 20 to 80 % in 20 min. Purified peptides were further analyzed on a Vydamass C18 100A 5μ 150 × 4.6 mm column, flow rate 1 mL min^{-1}; analyses were performed with the same gradient used for purification. Peptides were characterized by mass spectrometry on a Thermo Scientific LCQ Fleet ion trap. Purified peptides were lyophilized three times, the first to eliminate HPLC solvents, the second from a solution 6/4 (*v/v*) H_2O /CH_3COOH and the third in water.

Broth microdilution with TB peptide analogs exposed to harsh conditions

To investigate the tolerance of TB_KKG6K and D-Lys_TB_KKG6K to high temperature, extreme pH, proteolytic degradation, serum and cations, the peptides were exposed to respective experimental conditions and their IC_{90} against *C. albicans* was determined in broth microdilution assays. The thermal tolerance was investigated by heating the peptides in 0.05 × PDB to 95°C for 1 h, followed by cooling on ice for 10 min. The pH tolerance was tested by exposing the peptides to 25 mM glycine-HCl, pH 1.5 and glycine-NaOH, pH 11, respectively, for 24 h at 25°C. The peptide-buffer mixtures were subsequently diluted in 0.05 × PDB to reach the appropriate test concentrations at the standard pH of 5.6. The cation sensitivity of the peptides was assayed by supplementing 0.05 × PDB with increasing concentrations of $CaCl_2$ (1.5-6 mM, *w/v*), $MgCl_2$ (0.75-3 mM, *w/v*), NaCl (50-200 mM, *w/v*) and KCl (3-12 mM, *w/v*), respectively. The serum tolerance of the TB peptide

analogs was evaluated by supplementing 0.05 × PDB with increasing concentrations (1.25-5%, *v/v*) of heat-inactivated FCS (Merck Millipore, Burlington, MA, USA). The sensitivity to proteolytic degradation was evaluated by exposing each peptide to pepsin, trypsin and chymotrypsin, respectively, in a peptide: protease molar ratio of 30:1 for 3 h at 25°C in 0.05 × PDB. Then, pepsin and chymotrypsin were thermally inactivated at 100°C for 15 min. For the inactivation of trypsin, 1.25% FCS (*v/v*) was added to the trypsin-peptide solution. The peptides with inactivated proteases were then used in broth microdilution assays. The controls used in this experiment were the proteases and the peptides with and without exposure to protease inactivation conditions, respectively.

Cell death staining with propidium iodide

C. albicans (1×10^6 mL^{-1}) was incubated in 0.05 × PDB with 32 × IC_{90} D-Lys_TB_KKG6K in the presence or absence of 1 mM ascorbic acid (Sigma-Aldrich, St. Louis, MO, USA) for 4 h, respectively. This was followed by co-staining with 5 µg mL^{-1} PI (Sigma-Aldrich, St. Louis, MO, USA) for 10 min, after which microscopic analysis was conducted. Untreated *C. albicans* cells were used as a negative PI staining control. Microscopic imaging was performed with a fluorescence microscope (Axioplan, Carl Zeiss, Oberkochen, Germany), equipped with an AxioCam MR3 camera (excitation/emission filters at 565/620 nm for red fluorescence, Carl Zeiss, Oberkochen, Germany). Image processing and editing was done with Axiovision (Carl Zeiss, Oberkochen, Germany), GIMP (GNU Image Manipulation Program, version 2.8.20; www.gimp.org) and Microsoft Power Point (Microsoft Corp., Redmond, WA, USA).

Hemolytic potential

The hemolytic potential of the TB_KKG6K and D-Lys_TB_KKG6K was evaluated on Columbia blood agar plates (VWR, Radnor, PA, USA). Sterile filter discs (Ø 6 mm) were placed on the agar plates and loaded with 10 µL aliquots containing 50 µg of the peptides, or sterile water and 20% (*v/v*) Triton X-100 as negative and positive control, respectively. The plates were then incubated at 37°C for 24 h before evaluation. This experiment was performed twice.

Determination of the metabolic activity in primary human keratinocytes

To test the impact of the TB peptide analogs on the metabolic activity of primary human keratinocytes in vitro a colorimetric XTT assay was performed. Primary human keratinocytes were isolated from trunk skin biopsies and cultured in CellnTec basal medium (Table S2) [1]. One hundred µL of cells (2×10^4 mL^{-1}) per well were seeded into a flat-bottom 96-well microtiter plate (Nunclon Delta, Thermo Fisher Scientific, Waltham, MA, USA) and cultivated at 37°C and 5% (*v/v*) CO_2 until they reached 80% confluency. The growth medium was replaced with 100 µL of peptide solution prepared in the cell culture medium. Samples exposed to 50% ethanol (*v/v* in cell culture media) served as the positive control. For the negative control, 100 µL of cell culture media was added. For blank absorbance readings, wells with 100 µL of the respective media without cells were included. The plates were then incubated at 37°C, 5% (*v/v)* CO_2 for 16 h. Subsequently, 1 mg mL^{-1} XTT (Cell Signaling Technology, Danvers, MA, USA) and 1.53 mg ml^{-1} phenazine methosulfate (Cell Signaling Technology, Danvers, MA, USA) were mixed, and 50 µL was added per well. The plates were further incubated (37°C, 5% (*v/v*) CO_2) in the dark. The reduction of the tetrazolium dye by metabolically active cells was determined by measuring the absorbance at the wavelength 450 nm with a multi-mode microplate reader (FLUOstar Omega, BMG Labtech, Ortenberg, Germany) in an incubation time course of 1 - 6 h. All samples were prepared in triplicates and the assay was repeated at least twice.

Quantification of the cytokine IL-1α

The release of the pro-inflammatory biomarker IL-α by cells of the Phenion® OS-REp model (Henkel, Düsseldorf, Germany) was quantified in undiluted culture medium using an enzyme-linked immunosorbent assays (ELISA). The human IL-1 alpha ELISA Kit (Abcam, Cambridge, UK) was used according to the manufacturer's protocol. Mean concentrations (pg mL^{-1}) ± SD were calculated from three biological replicates per treatment and technical duplicates in the ELISA.

Supplementary Tables

Table S1. Microorganisms used in this study.

Microorganism	Specification	Source
Candida albicans	fluconazole sensitive	CBS 5982
Candida albicans 27700	fluconazole resistant	[2]
Candida glabrata		CBS 138
Candida parapsilosis		CBS 604
Staphylococcus aureus		ATCC 25923

Table S2. Media used in this study.

Medium	Composition[1]/Company[2]
CellnTec basal medium	CellnTec, Bern, Switzerland
Dulbecco's phosphate buffered saline (D-PBS)	0.1% (*w/v*) calcium chloride, 0.2% (*w/v*) potassium chloride, 0.1% (*w/v*) magnesium chloride hexahydrate, 8% (*w/v*) sodium chloride, 2.16% (*w/v*) sodium hydrogen phosphate
Phenion® OS_REp Air-Liquid interface medium	Henkel, Düsseldorf, Germany
Potato dextrose broth (PDB)	
Potato dextrose agar (PDA)	Potato dextrose broth, 2% (*w/v*) agar
Tryptic soy broth (TSB)	17 % (*w/v*) casein peptone, 3% (*w/v*) soy peptone, 5% (*w/v*) sodium chloride, 2.5% (*w/v*) dipotassium hydrogen phosphate
Tryptic soy agar (TSA)	Tryptic soy broth, 2% (*w/v*) agar

[1]Percent values are given as weight per volume (*w/v*) for solids and volume per volume (*v/v*) for solutions.

[2]If not otherwise stated, the chemicals and compounds were purchased from Sigma-Aldrich, St. Louis, MO, USA.

Table S3. The impact of protease inactivation conditions on the activity of the TB peptide analogs against *C. albicans*[§].

Compounds and Conditions	Growth [%]
Untreated control; standard conditions	100 ± 2.8
Untreated control; 1.25 % FCS	94.8 ± 4.3
**3.6 µM peptide + 0.125 µM protease**	
TB_KKG6K + Trypsin + 1.25 % FCS	104.8 ± 8.2*
D-Lys_TB_KKG6K + Trypsin + 1.25 % FCS	0.0 ± 0**
TB_KKG6K + Chymotrypsin + 100 °C, 15 min	75.4 ± 6.6*
D-Lys_TB_KKG6K + Chymotrypsin + 100 °C, 15 min	0.0 ± 0.1**
TB_KKG6K + Proteinase K + 100 °C, 15 min	83.7 ± 1.2*
D-Lys_TB_KKG6K + Proteinase K + 100 °C, 15 min	0.0 ± 0**
**0.125 µM protease alone**	
Trypsin + 1.25 % FCS	136.7 ± 14*
Chymotrypsin + 100 °C, 15 min	76.1 ± 6.9*
Proteinase K + 100 °C, 15 min	86 ± 4.5*
**3.6 µM peptide**	
TB_KKG6K + 100 °C, 15 min	0.0 ± 0.1**
D-Lys_TB_KKG6K + 100 °C, 15 min	3.05 ± 0.2**
TB_KKG6K + 1.25 % FCS	0.0 ± 0**
D-Lys_TB_KKG6K + 1.25 % FCS	0.0 ± 0**

[§]Broth microdilution assays were carried out with *C. albicans*. The concentration of the TB peptide analogs and the proteases represent the amount of compounds applied in a peptide: protease ratio of 30:1 and resulting in *C. albicans* growth ≤ 10% when compared to the growth control (assigned 100% growth) under standard assay conditions. Values shown represent the mean ± SD (n = 3); Significant differences between the treatments and respective growth controls are indicated; *$P \leq 0.05$, **$P \leq 0.005$.

Supplementary Figures

Figure S1

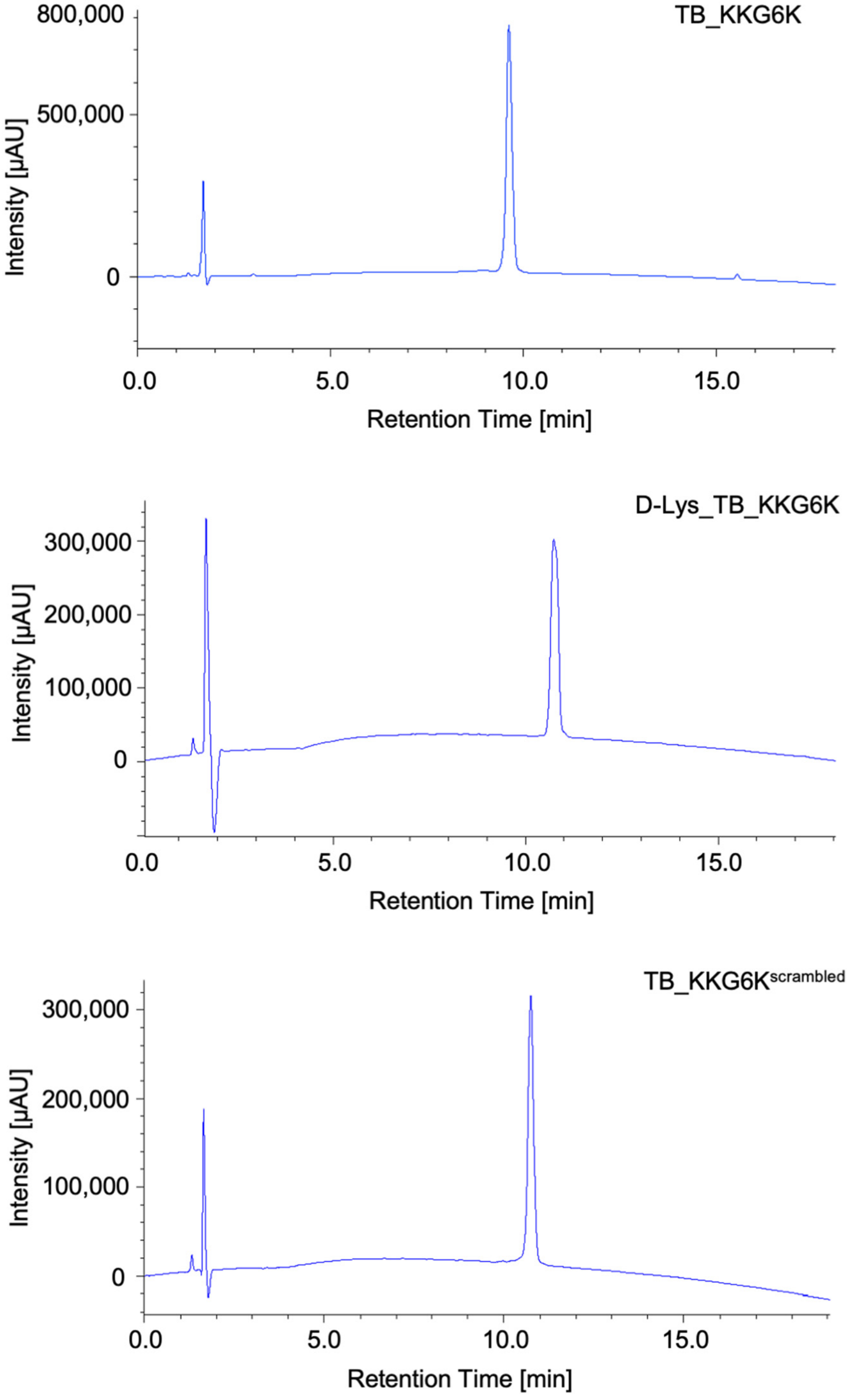

Fig. S1. RP-HPLC profiles of pure TB peptide analogs. The LC profiles were followed by monitoring the absorbance at 210 nm.

Figure S2

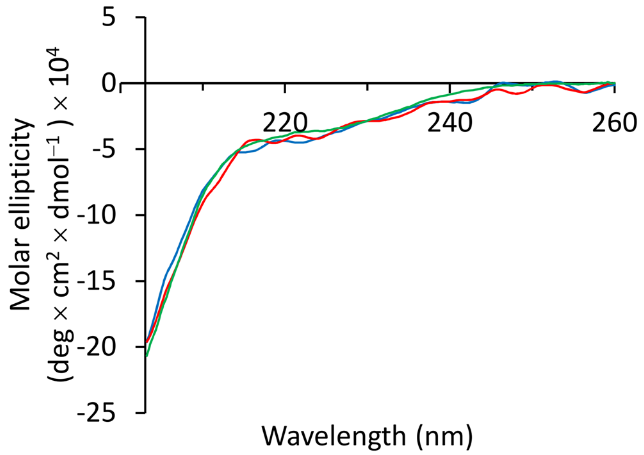

Fig. S2. CD spectra of TB peptide analogs. CD spectra of TB_KKG6K (blue), D-Lys TB_KKG6K (red) and TB_KKG6K^{scrambled} (green), recorded in 10 mM PBS (pH 7.4).

Figure S3

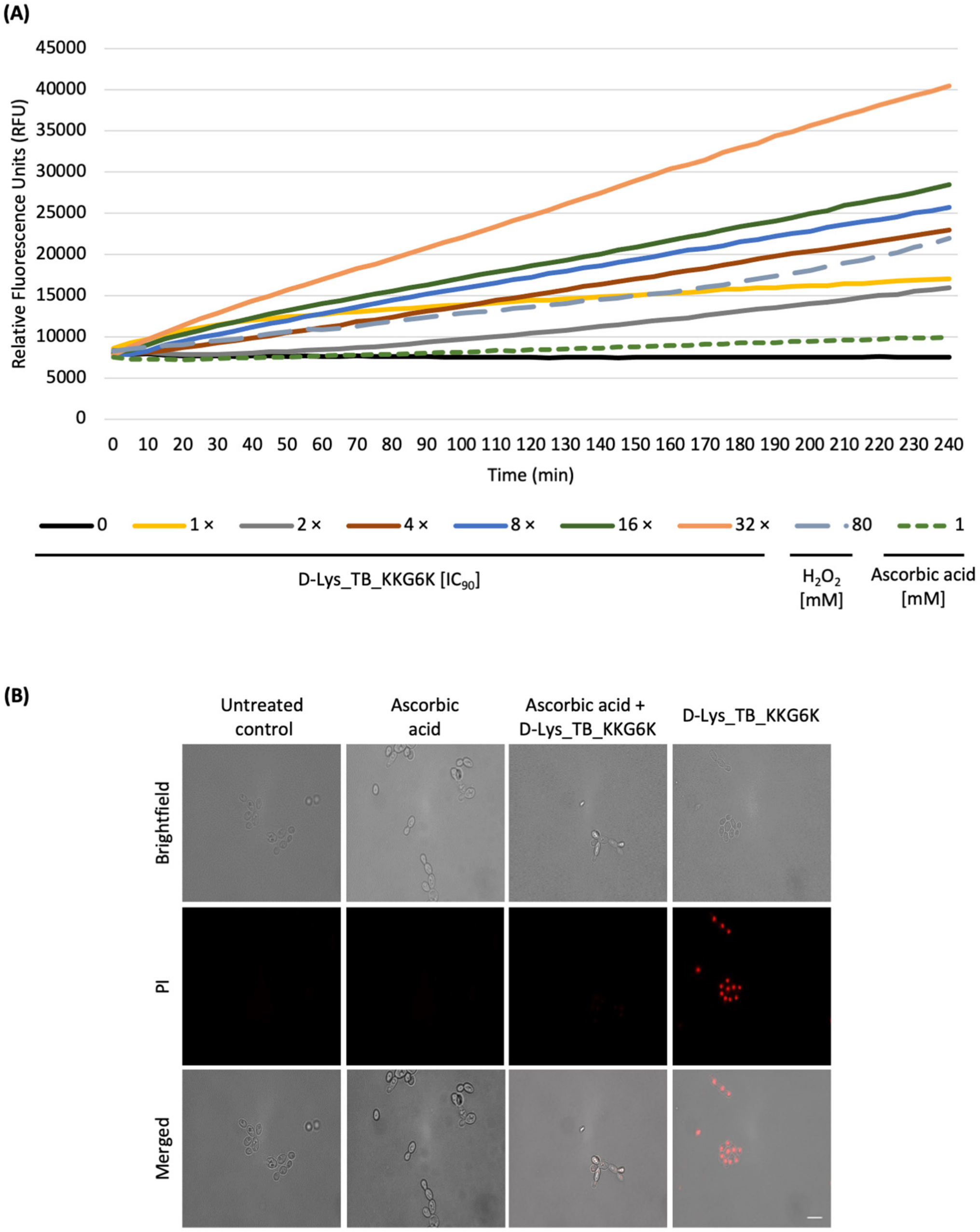

Fig. S3. Induction of iROS in *C. albicans* after treatment with D-Lys_TB_KKG6K monitored over a time period of 4 h. (A) The increase in the DCF fluorescence signal (represented in RFU) in *C. albicans* treated with 1 × IC_{90} - 32 × IC_{90} (1.8-57.6 μM) of D-Lys_TB_KKG6K in 0.5 × PDB at 30°C over a time period of 4 h. The negative control was not exposed to D-Lys_TB_KKG6K (0, black line). The effect of 80 mM H_2O_2, used as an iROS-inducing agent, is presented by a grey, dashed line. The prevention of iROS induction in *C. albicans* exposed to 32 × IC_{90} D-Lys_TB_KKG6K by the addition of 1 mM ascorbic acid is represented by a green, dotted line. (B) *C. albicans* was incubated in 0.05 × PDB at 30°C for 4 h with 1 mM ascorbic acid or with 32 × IC_{90} D-Lys_TB_KKG6K or in combination of these two compounds. This was followed by co-staining with PI for 10 min, after which microscopic analysis was conducted. Untreated *C. albicans* cells were used as a negative PI staining control. Scale bar, 5 μm.

Figure S4

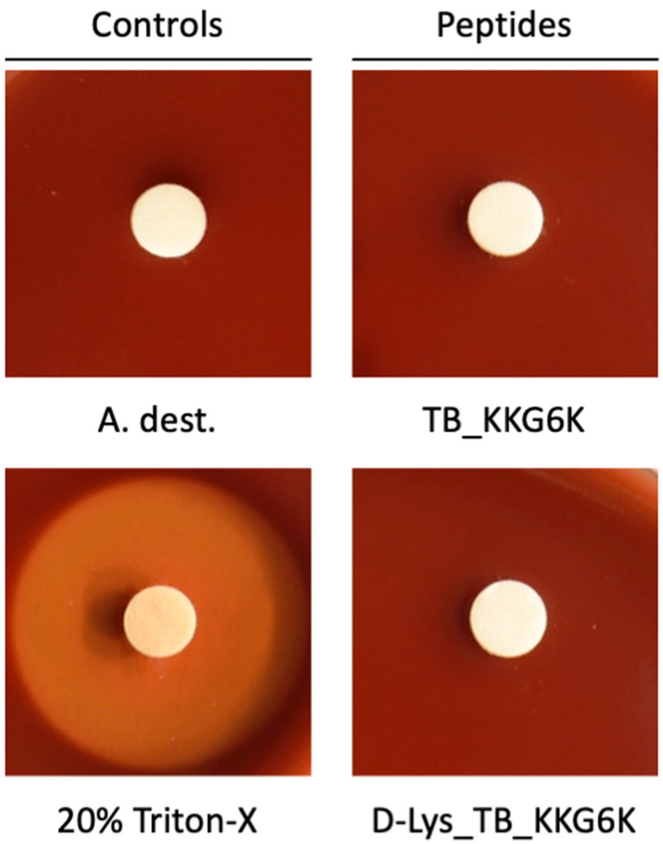

Fig. S4. Hemolytic activity of the TB peptide analogs. Sterile paper filter discs (Ø 6 mm) were placed on Columbia blood agar plates and 10 µL (50 µg) of TB_KKG6K and D-Lys_TB_KKG6K were applied on the discs, respectively. Positive (20% (*v/v*) Triton-X 100) and negative controls (A. dest.) were applied in the same volume. The plates were then incubated for 24 h at 37 °C.

Figure S5

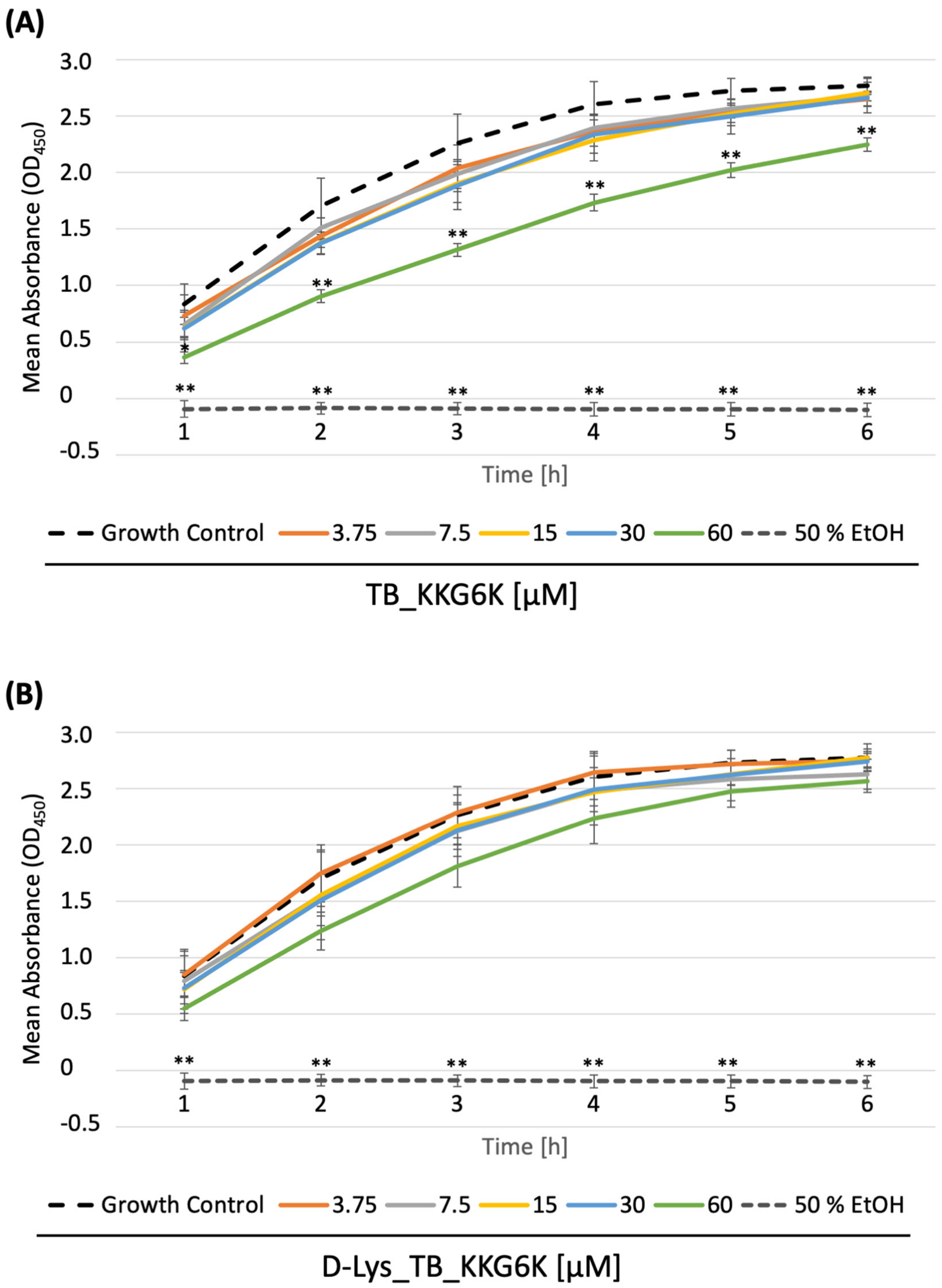

Fig. S5. Cellular metabolic activity of keratinocytes in response to increasing concentrations of TB peptide analogs over a time period of 6 h. (A) TB_KKG6K and (B) D-Lys_TB_KKG6K. The metabolic activity values shown represent the mean absorbance at 450 nm (OD_{450}) ± SD (n = 3). Significant differences between the treated samples and the negative growth control are indicated; $^{*}P \leq 0.05$, $^{**} P \leq 0.005$.

References

1. Blunder, S.; Rühl, R.; Moosbrugger-Martinz, V.; Krimmel, C.; Geisler, A.; Zhu, H.; Crumrine, D.; Elias, P.M.; Gruber, R.; Schmuth, M.; Dubrac, S. Alterations in epidermal eicosanoid metabolism contribute to inflammation and impaired late differentiation in *FLG*-mutated atopic dermatitis. *J. Invest. Dermatol.* **2017**, *137*, 706-715, doi:10.1016/j.jid.2016.09.034.
2. Kovács, R.; Holzknecht, J.; Hargitai, Z.; Papp, C.; Farkas, A.; Borics, A.; Tóth, L.; Váradi, G.; Tóth, G.K.; Kovács, I.; Dubrac, S.; Majoros, L.; Marx, F.; Galgóczy, L. In vivo applicability of *Neosartorya fischeri* antifungal protein 2 (NFAP2) in treatment of vulvovaginal candidiasis. *Antimicrob. Agents Chemother.* **2019**, *63*, e01777-18, doi:10.1128/AAC.01777-18.
